# Three-dimensional flow in Kupffer’s Vesicle

**DOI:** 10.1007/s00285-016-0967-7

**Published:** 2016-01-29

**Authors:** T. D. Montenegro-Johnson, D. I. Baker, D. J. Smith, S. S. Lopes

**Affiliations:** 1 Department of Applied Mathematics and Theoretical Physics, University of Cambridge, Cambridge, UK; 2 School of Mathematics, University of Birmingham, Birmingham, UK; 3 NOVA Medical School Faculdade de Ciências Médicas, Chronic Diseases Research Centre, CEDOC, Universidade Nova de Lisboa, Campo Mártires da Pátria, 130, 1169-056 Lisboa, Portugal

**Keywords:** Symmetry-breaking flow, Kupffer’s Vesicle, Cilia, Zebrafish embryo, 92C35, 76Z05, 92C15

## Abstract

Whilst many vertebrates appear externally left-right symmetric, the arrangement of internal organs is asymmetric. In zebrafish, the breaking of left-right symmetry is organised by Kupffer’s Vesicle (KV): an approximately spherical, fluid-filled structure that begins to form in the embryo 10 hours post fertilisation. A crucial component of zebrafish symmetry breaking is the establishment of a cilia-driven fluid flow within KV. However, it is still unclear (a) how dorsal, ventral and equatorial cilia contribute to the global vortical flow, and (b) if this flow breaks left-right symmetry through mechanical transduction or morphogen transport. Fully answering these questions requires knowledge of the three-dimensional flow patterns within KV, which have not been quantified in previous work. In this study, we calculate and analyse the three-dimensional flow in KV. We consider flow from both individual and groups of cilia, and (a) find anticlockwise flow can arise purely from excess of cilia on the dorsal roof over the ventral floor, showing how this vortical flow is stabilised by dorsal tilt of equatorial cilia, and (b) show that anterior clustering of dorsal cilia leads to around 40 % faster flow in the anterior over the posterior corner. We argue that these flow features are supportive of symmetry breaking through mechano-sensory cilia, and suggest a novel experiment to test this hypothesis. From our new understanding of the flow, we propose a further experiment to reverse the flow within KV to potentially induce *situs inversus*.

**Electronic supplementary material:**

The online version of this article (doi:10.1007/s00285-016-0967-7) contains supplementary material, which is available to authorized users.

## Nodal cilia and symmetry-breaking flow

In vertebrate embryos, the dorsal-ventral (back-front) and anterior–posterior (head-toe) axes are the first to be established (Hirokawa et al. 2009). The final left-right axis then needs to be chosen in a consistent manner in order to allow for asymmetric arrangement of internal organs. In humans for example, proper establishment of the left-right axis leads to a heart on the left and a liver on the right, despite apparent external bilateral symmetry. In certain species, the establishment of left-right asymmetry is governed by a structure called the node, first discovered in mice by Sulik et al. (1994). The fluid-filled node expresses ‘nodal’ cilia, which whirl in a clockwise direction when viewed from tip to base. These cilia generate a fluid flow which plays a key role in vertebrate left-right symmetry breaking (Nonaka et al. 1998).

Early theoretical and experimental studies of symmetry-breaking flow focused on the mouse node. The embryonic mouse node is a triangular depression, covered with a membrane and filled with fluid. The floor of the node is populated by the whirling cilia which generate the internal fluid flow. Flow in the mouse node was first modelled by Cartwright et al. (2004), who represented the motion of these cilia by point torques driving an infinite fluid. When cilia were tilted in the established posterior direction, clockwise whirling motion resulted in a directional leftward flow, thereby breaking left-right symmetry. The predicted tilt was subsequently observed experimentally by Okada et al. (2005). Other studies used time-dependent cilium models (Smith et al. 2007, 2008), showing that particles exhibit a leftward ‘loopy drift’ when released just above cilia. Upon reaching the left side of the node, particles recirculate slowly to the right just below the upper membrane.

**Fig. 1:**
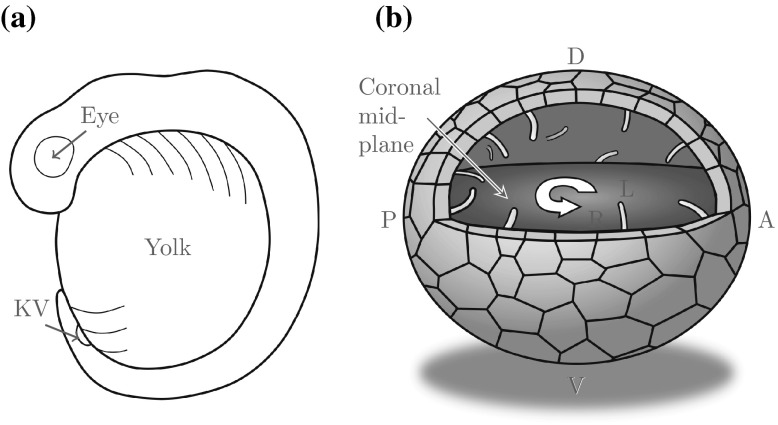
**a** Schematic of a zebrafish embryo, showing the approximate location of KV squeezed between the posterior and the yolk sac. **b** Cilia populate the inner surface of KV randomly, with a greater number on the dorsal roof and the highest density in the anterior-dorsal corner (Kreiling et al. 2007, redrawn). These cilia drive an anticlockwise vortical flow in the coronal midplane (midway between the dorsal and ventral poles)

In the wake of experimental interest (Kawakami et al. 2005; Kreiling et al. 2007; Okabe et al. 2008; Supatto et al. 2008), later studies began to examine the organising structure in zebrafish (Fig. 1a), known as Kupffer’s Vesicle (KV). KV is a transient structure that starts to form 10 hours post fertilisation (h.p.f.), and when fully formed at 14 h.p.f. (the 10 somite stage) its architecture is more complex than that observed in many species including mouse. In live embryos it is approximately spherical, around $50\,\upmu {\mathrm {m}}$ across, and its entire inner surface is populated by cilia which drive an internal flow (Fig. 1b). These cilia are not uniformly distributed; there are more cilia on the dorsal roof than the ventral floor, with the distribution most dense (clustered) in the anterior-dorsal corner (Kreiling et al. 2007). In wildtype fish, around a fifth of these cilia are immotile (Sampaio et al. 2014). The flow in the coronal midplane of KV is an anticlockwise vortex when viewed from the dorsal roof, with a centre displaced towards the anterior corner (Supatto et al. 2008), though the fully three-dimensional nature of the flow remains unknown.

Smith et al. (2012) modelled flow in KV using the regularised stokeslet boundary element method (Cortez et al. 2005; Smith 2009), incorporating a time-dependent computational mesh of the full geometry and cilia. Flow in the coronal midplane was calculated at each timestep over 5 cilium beats and then time averaged for comparison with experimental results (Supatto et al. 2008): a numerically intensive procedure taking approximately a day of runtime. It was found that dorsal tilt of equatorial cilia was required to best reproduce the experimentally-observed midplane flow. This model was extended by Sampaio et al. (2014) to account for natural variation in cilium length, number, frequency and distribution in and between embryos. The pattern of coronal flow was shown to be more robust in KV with higher numbers of cilia.

However, it is still unknown how contributions from individual tilted cilia in different locations on the inner surface of KV sum to produce a 3D flow field. As such, the mechanism by which this flow breaks left-right symmetry remains unclear: (a) differential release/absorption of morphogens or (b) mechano-sensory cilia. To address these questions a fully three-dimensional description of the flow is required. In this work, we combine the point torque modelling of Cartwright et al. (2004) with a boundary element mesh of the surface of KV, creating a hybrid singularity method. This approach bears favourable quantitative comparison with time-resolved modelling and experimental observations of flow speed and direction (Sampaio et al. 2014), and is able to quickly evaluate the three-dimensional flow within KV.

## Modelling KV and nodal cilia

### Fluid mechanics of stokes flow

In KV, cilia are around $5\,\upmu {\mathrm {m}}$ long, beating at around 30 Hz in a roughly conical envelope with a semi-cone angle of around 30$^\circ$. Taking the highest velocity *U* as approximately the length traced out by the cilium tip in a beat multiplied by the frequency, $U \approx 2\pi \cdot 5\sin {30}/(1/30) \approx 470\,\upmu {\mathrm {m}}\,{\mathrm {s}}^{-1}$, we see that the Reynolds number ${\mathrm {Re}}$ for flow in KV is

1 $$\begin{aligned} {\mathrm {Re}} = \frac{\rho U L}{\mu } \approx \frac{10^{3} \times 470\times 10^{-6}}{10^{-3}}\times 5\times 10^{-6} \approx 0.0024 \ll 1, \end{aligned}$$

where $\mu ,\rho$ are the dynamic viscosity and density of water respectively. Since the Reynolds number is small, fluid flow driven by nodal cilia may be modelled by the Stokes flow equations

2 $$\begin{aligned} \mu \nabla ^2{\mathbf {u}} -\nabla p + {\mathbf {F}} = 0, \quad \nabla \cdot {\mathbf {u}}=0, \end{aligned}$$

for ${\mathbf {u}}$ the fluid velocity, *p* the pressure and ${\mathbf {F}}$ body forces acting on the flow (Kim and Karrila 1991).

We will model the time-averaged whirling of a nodal cilium by a stationary point torque or “rotlet” $R_i(\hat{{\mathbf {n}}},{\mathbf {x}},{\mathbf {y}})$ in Stokes flow, which generates the flow field ${\mathbf {u}}({\mathbf {x}})$ (Blake and Chwang 1974)

3 $$\begin{aligned} u_i({\mathbf {x}}) = M R_i\left( \hat{{\mathbf {n}}},{\mathbf {x}},{\mathbf {y}}\right) = M\frac{\varepsilon _{ijk}\hat{n}_jr_k}{8\pi \mu r^3},\quad r_i = x_i - y_i,\quad r^2 = r_1^2 + r_2^2 + r_3^2, \end{aligned}$$

where ${\mathbf {y}}$ is the location, *M* is the strength, and $\hat{{\mathbf {n}}}$ is the unit normal direction of the rotlet. Because the Stokes flow Eq. (2) are linear, the flow from *n* identical cilia at locations ${\varvec{\chi }}^n$ is then simply the sum of the individual contributions

4 $$\begin{aligned} {\mathbf {u}}({\mathbf {x}}) = M\sum \limits _{n=1}^{N} {\mathbf {R}}\left( \hat{{\mathbf {n}}}^n,{\mathbf {x}},{\varvec{\chi }}^n\right) . \end{aligned}$$

In order to enforce the no-slip condition on the inner surface of KV, we also require an integral (Pozrikidis 1992) of the wall tractions ${\mathbf {f}}$ over the boundary *D*

5 $$\begin{aligned} {\mathbf {u}}({\mathbf {x}}) = \underbrace{\int \limits _D {\mathbf {S}}({\mathbf {x}},{\mathbf {y}}) \cdot {\mathbf {f}}({\mathbf {y}})\, {\mathrm {d}}S_y}_{\mathrm{no-slip~wall}} + \underbrace{\phantom {\int \limits _D}M\sum \limits _{n=1}^{N} {\mathbf {R}}\left( \hat{{\mathbf {n}}}^n,{\mathbf {x}}, {\varvec{\chi }}^n\right) }_{{\mathrm{cilia}}}, \end{aligned}$$

where $S_{ij}$ is the stokeslet tensor

6 $$\begin{aligned} S_{ij}({\mathbf {x}},{\mathbf {y}}) = \frac{1}{8\pi \mu }\left( \frac{\delta _{ij}}{r} + \frac{r_i r_j}{r^3} \right) . \end{aligned}$$

The wall tractions ${\mathbf {f}}$ are unknowns, and are calculated through specifying zero velocity on the surface of KV and solving the matrix system arising from the discretisation of Eq. (5). The time-averaged velocity at any point ${\mathbf {x}}$ within KV is then found by evaluating Eq. (5) with these tractions. However, for this model to represent flow inside KV, the strength and direction of the rotlets *M* must be prescribed such that the flow field it generates matches the time-averaged flow field of a cilium whirling about the axis $\hat{{\mathbf {n}}}$. Thus, we now proceed with an analytical derivation for *M*, and a validation of this model.

### Point torque cilium strength

**Fig. 2:**
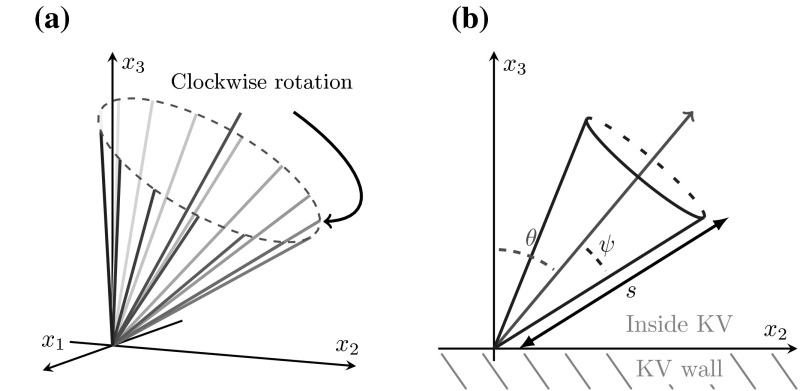
Nodal cilia. **a** A ciliary beat-pattern described by Eq. (7) with semicone angle $\psi = 30^{\circ }$ and tilt angle $\theta = 30^{\circ }$, demonstrating clockwise rotation when the cilium is viewed from tip to base. **b** Schematic demonstrating the tilt and semicone angles $\theta ,\psi$ respectively, showing the beat conical envelope and the measurement of the arclength *s*

Following the methodology of Smith et al. (2008), we consider a straight, tilted rod, tracing out a conical envelope (Fig. 2a), with centreline ${\varvec{\xi }}$,

7a $$\begin{aligned} \xi _1&= -s\sin \psi \cos \omega t, \end{aligned}$$

7b $$\begin{aligned} \xi _2&= -s\sin \psi \sin \omega t\cos \theta - s\cos \psi \sin \theta , \end{aligned}$$

7c $$\begin{aligned} \xi _3&= -s\sin \psi \sin \omega t \sin \theta + s\cos \psi \cos \theta . \end{aligned}$$

The parameter *s* is the arclength up the cilium, $\psi$ is the cilium semicone angle and $\theta$ is the angle at which the cilium is tilted towards the positive $x_2$ axis (Fig. 2b).

Using the local drag approximation of resistive force theory (Gray and Hancock 1955), the force ${\mathbf {f}}$ that the cilium exerts on the fluid is given by

8 $$\begin{aligned} f_j = C_\parallel \left[ \gamma \delta _{jk} - (\gamma - 1)\frac{\partial \xi _j}{\partial s} \frac{\partial \xi _k}{\partial s} \right] \frac{\partial \xi _k}{\partial t}, \end{aligned}$$

where

9 $$\begin{aligned} C_\parallel = \frac{4\pi \mu }{2\log (2q/a)},\quad C_\perp = \frac{8\pi \mu }{1 + 2\log (2q/a)},\quad \gamma = C_\perp /C_\parallel . \end{aligned}$$

The cilium diameter is given by *a*, and *q* is such that $a < q < L$ for cilium length *L*. The time-averaged moment per unit length that the cilium exerts on the fluid is then,

10 $$\begin{aligned} \langle {\varvec{\xi }} \wedge {\mathbf {f}} \rangle = C_\perp \omega s^2\sin ^2\psi \hat{{\mathbf {n}}}, \quad \hat{{\mathbf {n}}} = \cos \theta {\hat{{\mathbf {x}}}_3} + \sin \theta {\hat{{\mathbf {x}}}_2}. \end{aligned}$$

Integrating, the magnitude of the moment that the cilium exerts on the fluid is

11 $$\begin{aligned} M = \int \limits _0^L {C_\perp \omega s^2\sin ^2\psi \,{\mathrm {d}}s} = \frac{C_\perp \omega L^3\sin ^2\psi }{3}, \end{aligned}$$

which gives the strength of the equivalent rotlet. Thus, we can model the cilium-induced time-average flow at any given point ${\mathbf {x}}$ by a rotlet located at a point ${\mathbf {y}}$ of strength *M*

12 $$\begin{aligned} {\mathbf {u}}({\mathbf {x}}) = \frac{{\mathbf {M}}\wedge ({\mathbf {x}} - {\mathbf {y}})}{8\pi \mu |{\mathbf {x}} - {\mathbf {y}}|^3}, \quad {\mathbf {M}} = {M}\hat{{\mathbf {n}}} = \frac{C_\perp \omega L^3\sin ^2\psi }{3}\hat{{\mathbf {n}}} \end{aligned}$$

In Appendix 1, the consistency of this representation is demonstrated by showing that the equivalent rotlet over a plane boundary generates the same volume flow rate per beat as the full time-dependent model.

Since the volume flow rate of the equivalent rotlet is independent of its height above the boundary, we are free to choose its position in order to improve the near-field approximation of the time-averaged flow arising from a beating cilium. To achieve this, we consider a weighted average whereby the volume flow rate produced by portions of the cilium below the rotlet is equal to that above. The volume flow rate *Q* from a point force ${f_1}$ in the *x*-direction a distance *d* above a boundary is given by $Q\propto f_1 d$. In the resistive force theory approximation, $|f| \propto |u| \propto s$, and since $d\propto s$ then the volume flow rate per unit length is proportional to $s^2$,

13 $$\begin{aligned} \int \limits _0^{d_r} \frac{f_1(s) \xi _3(s)}{\pi \mu }\,{\mathrm {d}}s= & {} \int \limits _{d_r}^{L\cos \psi } \frac{f_1(s)\xi _3(s)}{\pi \mu }\,{\mathrm {d}}s \nonumber \\ \therefore \ \int \limits _0^{d_r} s^2\,{\mathrm {d}}s= & {} \int \limits _{d_r}^{L\cos \psi } s^2\,{\mathrm {d}}s \Rightarrow d = \frac{L\cos \psi }{\root 3 \of {2}} \approx 0.79L\cos \psi . \end{aligned}$$

In Appendix 1, we have compared the near-field flow of the equivalent rotlet with the time-averaged flow of a time-dependent regularised stokeslet model cilium. We find that in fact the best flow agreement is obtained for $d = 0.82L\cos \psi$; for just half a length away from the cilium, the relative error in the flow is less than 10 %. This error decays quickly with distance from the cilium, and the direction of the flow is consistent between both models. Furthermore, since cilium-induced volume flow rate is proportional to $L^3$, a 10 % difference in flow magnitude corresponds to a 3 % difference in the length of the cilium. Such natural variation in cilium length occurs in KV (Sampaio et al. 2014), accurate measurements of which are also subject to limitations. Thus, to gain insight into the nature of the flow within KV, we consider this level of accuracy acceptable.

### Numerical implementation

In order to model the structure of KV, we distribute rotlets within a boundary element mesh of a sphere. The strength of these rotlets is set to be equivalent to cilia of length $5\,\upmu {\mathrm {m}}$ beating at 30 Hz with a semicone angle of $30^{\circ }$. Rotlets are initially untilted, facing towards the centre of the sphere, and the sign of the strength represents clockwise rotation when viewed from the centre (ie, tip to base). Cilia are then tilted by a specified amount in the local dorsal direction (Smith et al. 2012).

Equation (5) is discretised over a spherical mesh of 512 quadratic triangular elements; the unknown traction is modelled as taking the constant value ${\mathbf {f}}[l]$ over each mesh element *E*[*l*] of the surface *D*, so that the discrete version of Eq. (5) is given by

14 $$\begin{aligned} u_j({\mathbf {x}}) = 0 = \sum \limits _{l=1}^{512}{f_i[l]}\int _{E[l]}S_{ij}({\mathbf {x}},{\mathbf {y}})\,{\mathrm {d}} S_y + M\sum \limits _{n=1}^{N} {R}_j\left( \hat{{\mathbf {n}}}^n,{\mathbf {x}},{\varvec{\chi }}^n\right) , \end{aligned}$$

for ${\mathbf {x}} \in E[l]$. The element tractions ${\mathbf {f}}[l]$ are found by solving the linear system (14), and are such that the velocity at the centroid of each element is zero. Once the linear system is solved, the element surface tractions can be used in the discrete form of Eq. (5) to calculate the fluid velocity at any point within the mesh. Since time-averaging of the cilia beat has been incorporated through the use of the equivalent rotlet, this matrix system need only be solved once, taking a few seconds of runtime in contrast to the time-dependent models where the matrix system is solved at each time-step.

The code is implemented in Fortran 90 (gfortran, GNU Compiler Collection), with mesh generation and boundary integrals performed using routines adapted from BEMLIB (Pozrikidis 2002). The linear system is solved by LU factorisation with the LAPACK routine dgesv. Flow visualisation is performed using custom routines in Matlab, with streamline data calculated using the second-order variable two-step Adams Bashforth method (Press 2007). Since to the authors’ knowledge there is no known general analytical solution for a rotlet inside a sphere, the method is verified against the solution for a stokeslet in a sphere (Oseen 1927; Maul and Kim 1994) for which we find a relative error in the flow speed of $<$1 % throughout the domain. We now proceed to analyse the flow in KV.

## Results

In the following results, the orientation of KV is consistent with Fig. 1b. In figures with streamlines, the vortex direction is transparent to opaque and denoted by the large 3D arrows. Flow speed is indicated by streamline colour, and a lighting effect is used to show the 3D shape. The positions of rotlets, representing cilia, are given by the grey spheres. The lighter rotlets are closer to the right hemisphere. For each streamline figure, a corresponding video is available in the online supplementary material.

### Placement of “useful” cilia

**Fig. 3:**
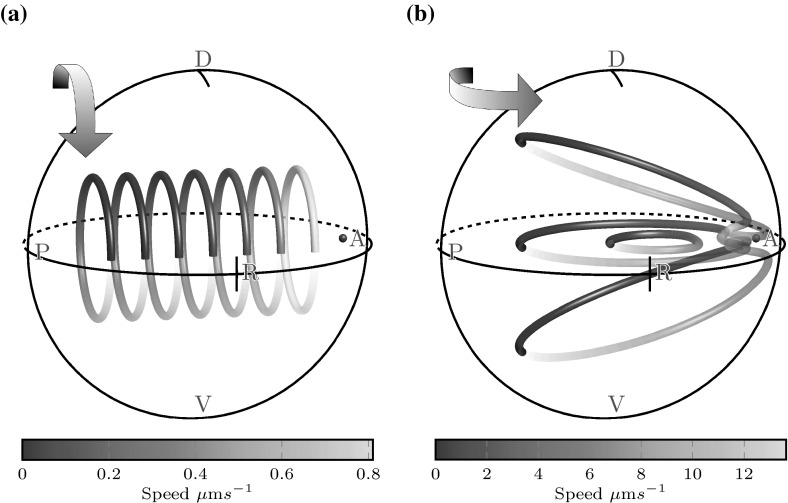
Flows from a single rotlet located at the anterior equator, viewed from the right side of KV. **a** An untilted rotlet, showing vortical flow throughout KV. **b** A $90^\circ$ tilt is applied to the rotlet, showing an anticlockwise vortex when viewed from the dorsal pole. By linearity of the Stokes flow equations, the flow arising from equatorial cilia tilted by $30^\circ$ is a linear combination of these two flows. See the supplementary material for the videos corresponding to these plots

**Fig. 4:**
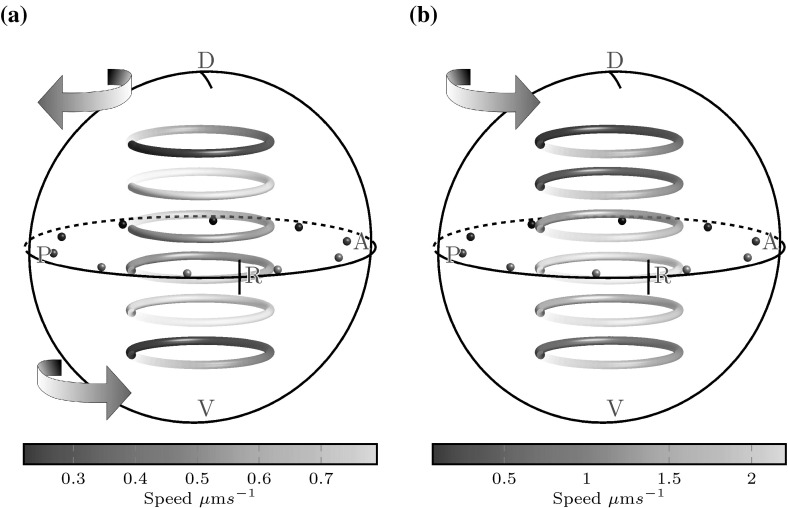
Flows from a ring of equatorial cilia viewed from the right side of KV, with the same format as Fig. 3. **a** Flow from an untilted ring of 10 equatorial cilia, showing an anticlockwise vortex in the ventral hemisphere (*bottom*) and a clockwise vortex in the dorsal hemisphere (*top*). **b** After these equatorial cilia are tilted $30^\circ$ to the dorsal pole, flow is an anticlockwise vortex throughout. See the supplementary material for the videos corresponding to these plots

We begin our analysis by considering a single cilium placed at the anterior equator, on the right side of each panel in Fig. 3. For an untilted cilium, the axis of the rotlet is perpendicular to the wall and flow is vortical (Fig. 3a); streamlines are concentric rings about the anterior–posterior axis. Velocity is constant on each streamline, denoted by the colour map, and decays as the distance away from the cilium increases. The direction of the vortex is clockwise when viewed from the posterior (left of figure), which as expected is the direction in which the cilium rotates. This flow immediately shows us that untilted cilia located on the dorsal roof (top of figure) are ‘useful’ (Smith et al. 2014) for generating the experimentally observed (Supatto et al. 2008) anticlockwise flow in the coronal midplane (midway between the dorsal and ventral axes). Conversely, cilia on the ventral floor are ‘antagonistic’ to this flow, as they generate an opposite whirlpool; a system comprising a single cilium at each of the dorsal and ventral poles would have zero flow in the coronal midplane. Since the Stokes flow equations (2) are linear, solutions may be superposed. Thus, in the absence of cilium tilt, a surplus of cilia on the dorsal roof is necessary to generate the observed anticlockwise flow.

**Fig. 5:**
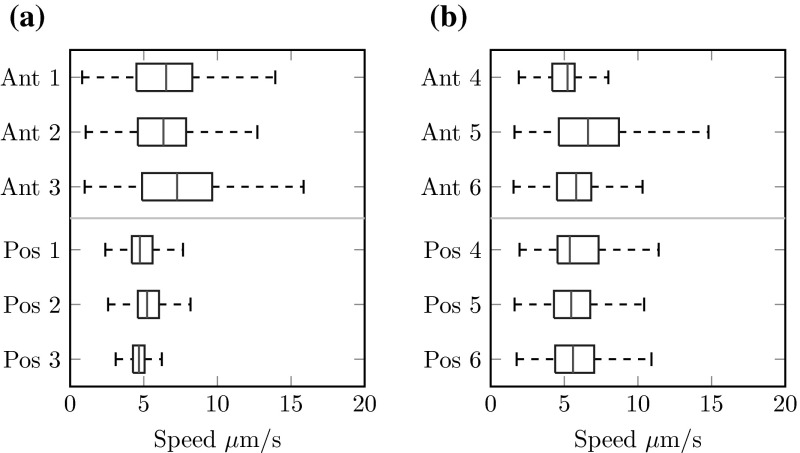
*Box plots* of velocity magnitude sampled at 1500 random points in the anterior third and posterior third of the coronal midplane with $r \le 17.5\,\upmu {\mathrm {m}}$, i.e. at least half a length from any equatorial cilia. **a** The velocity for three separate random placements of cilia following the Kreiling distribution (Kreiling et al. 2007), showing consistently higher velocity in the anterior. **b** The velocity for three further random placements of cilia without anterior clustering, i.e. with 80 % of cilia on the dorsal roof and 20 % on the ventral floor, showing equal velocity in the anterior and posterior

Untilted cilia on the dorsal roof contribute to the observed anticlockwise flow in the coronal midplane, but equatorial cilia which are tilted towards the dorsal pole also contribute. Figure 3b shows flow arising from a rotlet aligned in the dorsal-ventral direction, parallel to the wall, which generates an anti-clockwise vortex. Because solutions to Stokes flow can be superposed, the flow from a tilted rotlet, representing a tilted cilium, can be thought of as a linear combination of the flow in Fig. 3a, b. This contribution motivates consideration of a ring of equatorial cilia (Fig. 4). Such a ring of untilted cilia induces (when viewed from the dorsal roof) clockwise flow in the dorsal hemisphere, opposite to the naturally-occurring flow, and anticlockwise flow in the ventral hemisphere (Fig. 4a). Thus, equatorial cilia that are not tilted in the dorsal direction are in fact antagonistic to anticlockwise vortical flow in the ventral hemisphere. However, once these cilia are tilted by around $30^\circ$, similar to the average $26.6^\circ$ observed in mice (Nonaka et al. 2005), flow is anticlockwise flow throughout KV (Fig. 4b), so that dorsally tilted equatorial cilia strengthen the anticlockwise vortex.

### Natural cilium distribution and tilt

Before analysing the three-dimensional flow arising from a “natural” distribution of cilia, we draw comparisons with previous experimental data for wildtype (WT) embryos (Sampaio et al. 2014). We consider the coronal midplane flow generated by three random placements of 30 cilia sampled from the experimentally observed distribution of (Kreiling et al. 2007), where 20 % of cilia are found on the ventral floor, 17 % in the dorsal posterior corner, 25 % in the dorsal mid-section and 38 % in the anterior-dorsal corner. Cilia are tilted by an angle of $\theta = 30\sin (\alpha ), \alpha \in [0,180]$ degrees towards the dorsal pole, for $\alpha$ the cilium’s latitude between the dorsal and ventral poles. This way, equatorial cilia are tilted by $30^\circ$ and the degree of tilt smoothly decreases to zero at either pole.

Figure 5a shows a box plot of the flow speed at 1500 randomly selected points in the anterior and posterior thirds of the coronal midplane for three random natural placements of cilia. These boxplots show consistently higher velocities, at around 40 %, in the anterior when compared to the posterior, and bear a striking resemblance to the data of Sampaio et al. (Fig. 2b). In particular the median velocity we find is $7\,\upmu {\mathrm {m}}$/s in the anterior and $5\,\upmu {\mathrm {m}}$/s in the posterior are similar to the values of $9\,\upmu {\mathrm {m}}$/s in the anterior and $6\,\upmu {\mathrm {m}}$/s reported from averaging 675 particle tracks from 7 embryos. A likely explanation for the underestimation given by our code is that we have restricted ourselves to a central region of the flow, at least 2.5 lengths from any cilium where the approximation of a time-averaged flow is valid, whereas particle tracks in Sampaio et al. include near-cilium interactions where the flow velocity is much higher. Furthermore, since Sampaio et al. follow native particles within KV which originate at the surface, it is possible that a greater number of particles are tracked nearer to the surface where flow is stronger, whereas our sample is distributed evenly.

**Fig. 6:**
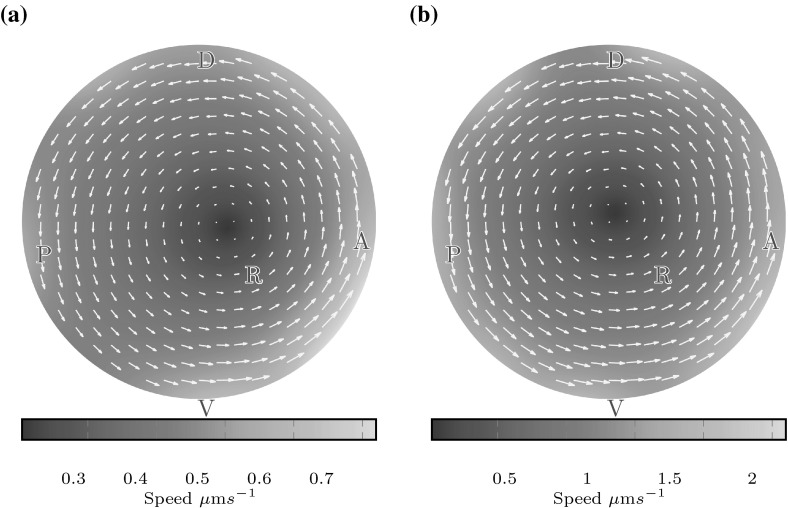
Flow speed in the coronal midplane for $r \le 15$ for **a** a natural distribution (dist 1), showing faster flow in the anterior (*right*) and a displaced vortex centre point, and **b** an unclustered distribution (dist 6), showing no significant anterior–posterior speed asymmetry and a vortex centre point located at the origin. A displaced centre point was observed experimentally by Supatto et al. (2008)

If the anterior clustering is disrupted, the difference between anterior and posterior flow speeds disappears. Figure 5b shows box plots of the flow speeds in the anterior and posterior thirds of the coronal midplane for three random cilium placements such that 20 % of cilia are on the ventral floor and 80 % are on the dorsal roof; as observed in nature, but without bias to the anterior-dorsal corner. Here we see no consistent difference between anterior and posterior flows; the median flow speeds are $5.5\,\upmu {\mathrm {m}}$/s in both the anterior and posterior. The effect of anterior clustering upon the flow is also shown in Fig. 6. Figure 6a shows midplane flow from a natural distribution with anterior clustering. Flow is faster in the anterior, and the centre point of the vortex is displaced to the anterior, as observed by Supatto et al. (2008). In contrast, whilst there remains an anticlockwise vortical flow for the unclustered distribution (Fig. 6b), the vortex centre point is no longer displaced.

**Fig. 7:**
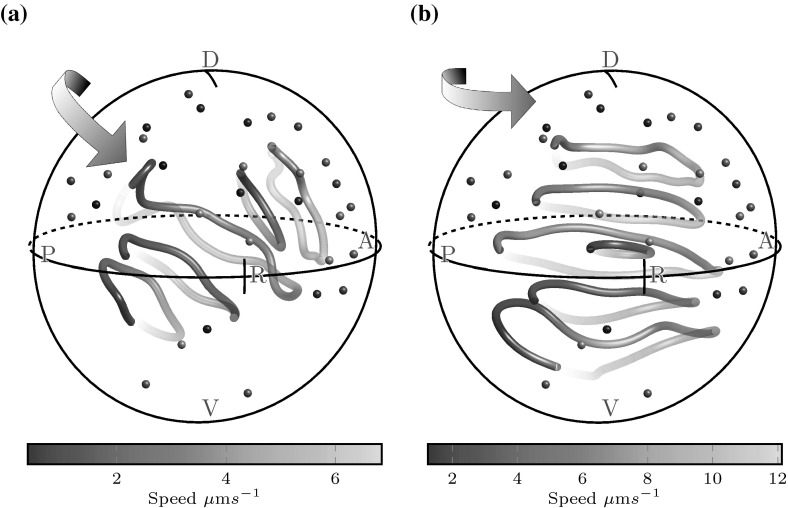
Flows from a natural, random distribution of cilia viewed from the right side of KV. **a** Untilted cilia following the distribution of Kreiling et al. (2007), showing an anticlockwise vortical flow about the anterior dorsal corner and **b** the same placement of cilia tilted to the dorsal pole, flattening this vortex. See the supplementary material for the videos corresponding to these plots

We now proceed to examine the three-dimensional flow arising from a natural distribution of 30 cilia, and the effects of dorsal tilt. For untilted cilia, flow is an anticlockwise vortex with a centreline pointing towards the anterior-dorsal cluster, the location of the majority of cilia (Fig. 7a). Such a flow would result in particles moving in and out of the coronal midplane, which is not observed in experiments. However, we then tilt the equatorial cilia towards the dorsal pole by an angle of $\theta = 30\sin (\alpha ), \alpha \in [0,180]$ degrees. Figure 7b shows that the effect of tilt is to flatten the vortical flow into coronal planes. The centreline of the vortex is now aligned with the *z*-axis, and the flow velocity is higher than in the untilted case because there is a greater excess of ‘useful’ cilia contributing to the final flow. Furthermore, flow velocities are consistently higher throughout the anterior hemisphere than the posterior hemisphere. These qualitative features were also consistent for simulations with 20 and 40 cilia.

## Proposed experiments

Based on these results, and our new understanding of how cilia generate the 3D flow-field in KV, we propose two novel experiments to (a) test the mechano-sensory hypothesis by disrupting the clustering of cilia in the anterior dorsal corner and (b) reverse the flow field to potentially induce *situs inversus*.

### Anterior declustering

The effect of anterior clustering of cilia is to increase the strength of the vortex in the anterior corner, where the vortical flow is travelling leftward. If a consistent vortex direction were sufficient to break symmetry, as might be expected from morphogen transport/absorption, our results suggest that anterior clustering would be unnecessary, provided a surplus of cilia were located on the dorsal roof. Thus it seems plausible that both the direction of the vortex and the relative strength between flow at the anterior and posterior edges of KV are important.

This observation motivates a novel experiment to examine the morphogen vs mechano-sensory hypotheses as mechanisms for symmetry breaking. By selectively knocking-out the motility of some cilia in the anterior cluster, perhaps through laser ablation, it is possible to achieve an unclustered distribution, with a dorsal surplus, of motile cilia within KV. These motile cilia would drive a global anticlockwise vortical flow without a consistent speed difference between the anterior and posterior hemispheres, as simulated in Fig. 6b. If such embryos were to exhibit significant situs defects, this would support the presence of a mechano-sensory component to symmetry-breaking, since for a pure morphogen transport mechanism the anticlockwise direction of the vortex should be sufficient. However, it should be noted that such a result would not rule out a morphogen-based component in symmetry-breaking, as discussed further in Sect. 5.

### Flow reversal

Since we now understand how some cilia can contribute to the vortical flow in KV, while others are antagonistic, we can furthermore conceive a non-invasive analogue of flow reversal experiments in mice (Nonaka et al. 2002) for zebrafish: through knocking-out the motility of ‘useful’ dorsal cilia leaving only the antagonistic ventral cilia. In mice, embryos that developed following flow reversal exhibited *situs inversus*, reversed positioning of internal organs. Thus, such an experiment might provide valuable further insight into the mechanism of symmetry breaking in zebrafish, particularly when taken together with the experiment suggested in Sect. 3.2. We now use our model to examine the feasibility of generating a clockwise flow in KV.

**Fig. 8:**
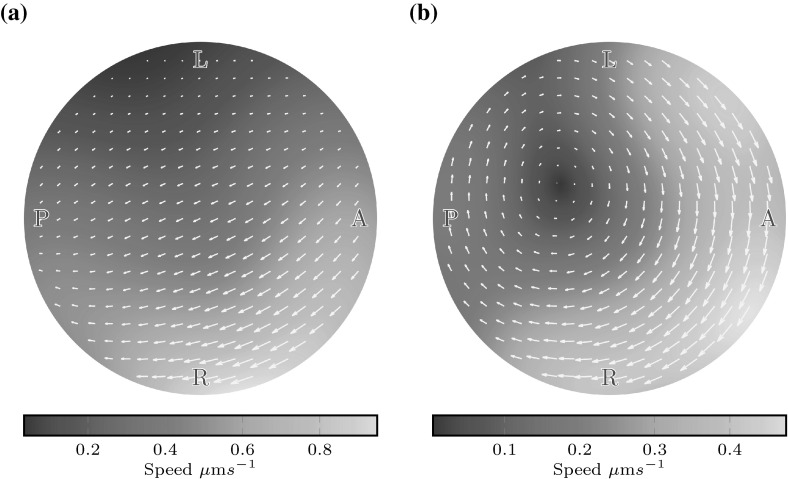
Coronal midplane flows from distributions of 40 cilia for which only cilia in the bottom ventral third are motile. **a** Flow in natural distribution 2, showing slight directional motion and **b** flow in an unclustered, random distribution such as those created by Wang et al. (2012) showing clockwise vortical flows in contrast with the naturally-occurring anticlockwise vortex (Fig. 6a)

Figure 8 shows two flow fields in the coronal midplane for simulated embryos where only the cilia in the ventral quarter of KV are motile. In Fig. 8a, natural distribution 2 shows a more directional than vortical flow in the majority of the midplane, which is clearly a poor reversal of the anticlockwise vortical flow (Fig. 6a). Since there are very few cilia on the ventral floor in the naturally-occurring cilium distribution, the location of these cilia are not robust to random variations in precise placement. Thus, it is difficult to ensure there are sufficient cilia in the correct location to ensure a global clockwise vortex.

However, Wang et al. (2012) showed that the process of differential cell length to width growth which leads to cilium clustering in KV can be disrupted by interfering with non-muscle myosin II activity. For an embryo where cilia are evenly distributed throughout KV, a greater number of cilia are found on the ventral floor, and the corresponding coronal midplane flow is a clockwise vortex (Fig. 8b). Thus, through either selecting wildtype embryos with a large number of ventral cilia, or by disrupting cilium clustering in the manner of Wang et al. (2012), it is possible to reverse the vortical flow in KV by knocking-out the motility of all cilia except those on the ventral floor.

Note that for the particular random distribution chosen in Fig. 8b flow is stronger in the anterior than the posterior. However, without clustering this effect is essentially random; whilst the procedure above could reverse the direction of flow in KV, it is not sufficient to ensure a reversal of the relative flow strength in the anterior and posterior hemispheres. Faster reversed flow in the anterior might be consistently possible, however, if a number of cilia in the ventral posterior corner were ablated, or if equatorial cilia were somehow made to tilt towards the ventral pole.

## Discussion

In this work, we have used a time-averaged singularity model that combines the methods of Cartwright et al. (2004) and Smith et al. (2011) to examine three-dimensional, symmetry-breaking flow in the zebrafish organising structure, KV. An analytical formula was derived to determine the strength of a point torque representing a cilium of given length, and at the optimum location this point torque was shown to accurately represent the time-averaged flow generated by a whirling cilium just half a length away. While this model is valid for examining flow throughout the majority of the volume of KV, the time-dependent nature of the beat becomes important when analysing the motion of suspended particles closer to the cilium. Rather than a constant streaming, such particles have been shown in mice to execute a ‘loopy drift’ (Smith et al. 2007, 2008). Furthermore, the time-averaged approach is not appropriate for studying dynamics in the chaotic layer between cilium tip and KV wall (Supatto et al. 2008). For such studies, the current model could be modified to include time-dependent slender body representations of cilia using the solution for a stokeslet within a sphere (Oseen 1927; Maul and Kim 1994).

The flow from a single cilium was examined; through linearity of Stokes flow and superposition of solutions, this flow demonstrated that a surplus of cilia on the dorsal roof alone was sufficient to generate the experimentally observed anticlockwise vortex. A ring of equatorial cilia was antagonistic to this flow in the dorsal half of KV when untilted, but when tilted in the dorsal direction added to the strength of the vortex. This analysis suggests that flow reversal in KV might be achieved experimentally through selectively “knocking-out” the motility of dorsal and equatorial cilia, as achieved for left-sided cilia by Sampaio et al. (2014). This hypothesis was tested, and was shown to be possible if the differential cell shape mechanism responsible for cilium clustering was also disabled, as in Wang et al. (2012). Simulated flow from natural distributions compared favourably with previous experimental data, and dorsal tilt was shown in this case to flatten the flow into a vortex about the dorsal-ventral axis.

What can this flow reveal about possible mechanisms of symmetry breaking in KV? Two possible mechanisms for vertebrate symmetry breaking have been posited: left-right differential concentration of morphogens responsible for breaking symmetry, and mechanical sensing of the flow direction and strength by cilia. To generate a non-uniform concentration of morphogen in KV, these must be introduced and then reabsorbed at the surface through endocytosis. Morphogens cannot be introduced with any inherent left-right asymmetry, and since the flow is vortical in coronal planes throughout KV, morphogens introduced at the dorsal and ventral poles would not be advected. Thus, any morphogen responsible for symmetry breaking would have to be introduced at either the anterior or posterior corners. Because flow is anticlockwise when viewed from the dorsal roof, morphogen introduced at the anterior would first travel past the left side of KV, whereas that introduced at the posterior would first pass the right. Thus, if morphogens are reabsorbed sufficiently quickly (i.e. before reaching the other side), a left-right differential concentration may be set up, thereby breaking symmetry.

However, it is not clear in this system why anterior clustering of cilia should be necessary, as the mechanism should be equally valid for the unclustered distributions in Figs. 5b and 6b. If higher velocity in the anterior corner relative to the posterior corner is indeed required, it is supportive of at least a mechano-sensory component to symmetry breaking; sensory cilia in the anterior would be deflected to the left more strongly than cilia in the posterior were deflected to the right. We proposed two experiments to control flow strength and direction in KV which could help in the systematic analysis of zebrafish symmetry breaking. (1) By selectively knocking-out the motility of a portion of cilia in the anterior cluster of a wildtype embryo, an unbiased anticlockwise vortical flow can be established. (2) By selectively knocking-out the motility of dorsal cilia in embryos with homogeneous cilia distributions, an unbiased clockwise vortical flow can be established. The results of such experiments would require careful interpretation, particularly in the event of there being both mechanical and morphogen-based elements to symmetry breaking, but could provide valuable additional insight when applied in conjunction with other genetic tests on mutant and knock-down embryos.

The numerical method presented is able to construct the time-averaged flow in KV quickly and efficiently. From this model we have been able to gain further understanding of the effects of individual cilia placement and tilt, and insight into the way these effects combine to give the full flow, which was not possible with two-dimensional visualisations. The nature of the three-dimensional flow motivates a experimental means of flow retardation and reversal, which may provide further evidence to the mechanism of symmetry breaking. The flexibility of describing the geometry with a boundary element mesh will further allow for embryo-specific flow analyses in which the shape of KV and cilia locations for any given embryo are extracted from imaging data. The simple analytical treatment of the cilium point torque strength suggests that this method may in addition be useful in investigating other fish species with complex organising structures.

### Electronic supplementary material

Below is the link to the electronic supplementary material.

Supplementary material 1 (avi 13496 KB)

Supplementary material 2 (avi 15467 KB)

Supplementary material 3 (avi 14458 KB)

Supplementary material 4 (avi 14650 KB)

Supplementary material 5 (avi 15342 KB)

Supplementary material 6 (avi 17027 KB)

## Figures and Tables

**Fig. 9:**
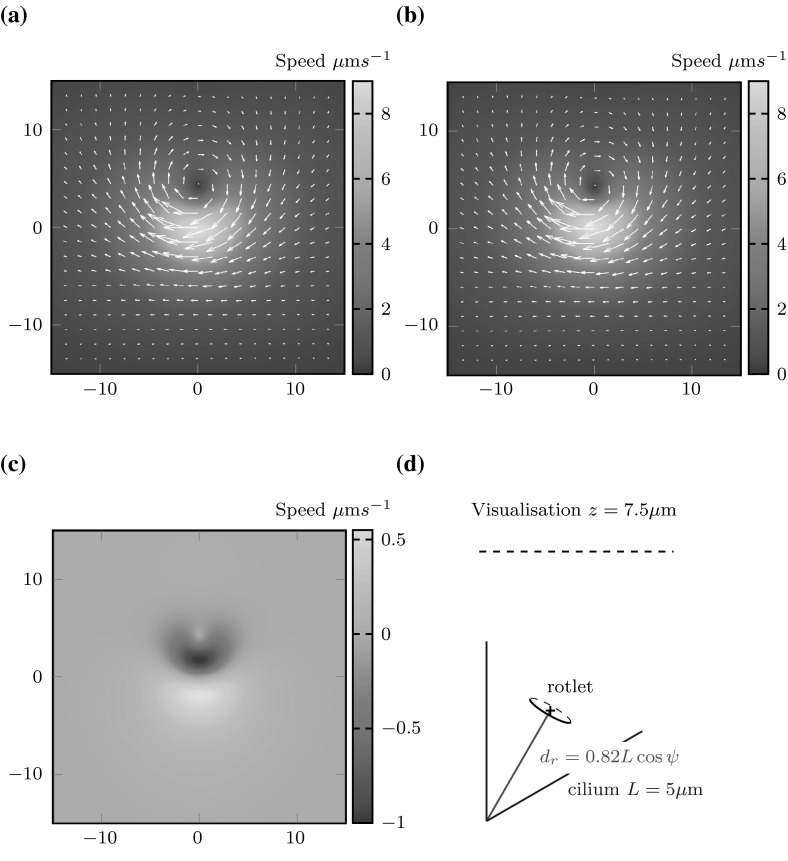
Comparing time averaged flow (**a**) of a $5\,\upmu {\mathrm {m}}$ with $\psi = 30^{\circ }, \theta = 30^{\circ }$ beating at 30 Hz with the equivalent rotlet (**b**), in the plane $z = 7.5\,\upmu {\mathrm {m}}$. Distances are shown in microns, and the colormaps in figures **a**–**c** are the speed of the flow in microns per second. Visual comparison of **a** and **b** show remarkable similarity between the two solutions, with **c** showing at worst a 10 % error in the speed calculated by the rotlet model, and very good qualitative agreement in flow direction and magnitude. Evaluation of the results in **a** took approximately 2 h on a laptop computer, whereas **b** took approximately 10 s. **d** Shows the envelope of the cilium, the location of the equivalent rotlet, and the location of the plane in which the velocity is evaluated

**Fig. 10:**
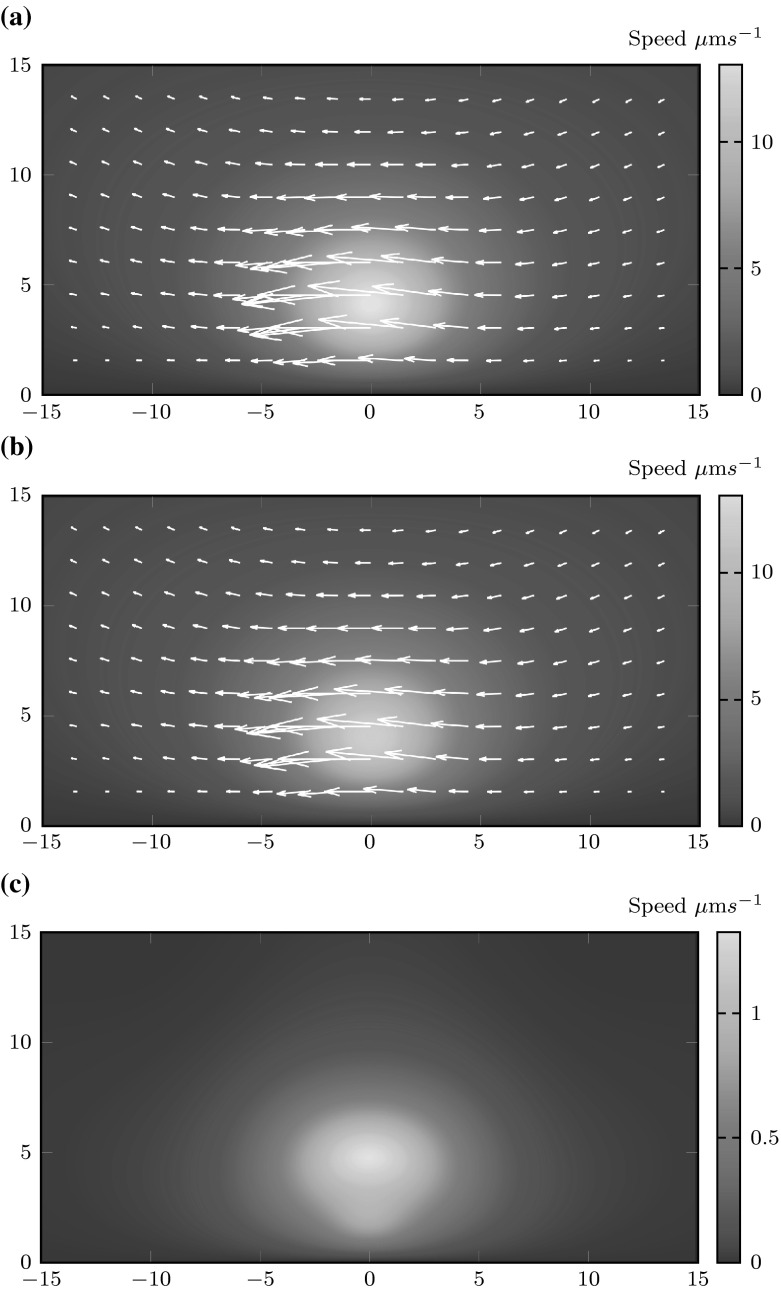
Time averaged flow of the same cilium (**a**) and equivalent rotlet (**b**) as in Fig. 9 in the plane $y = -2.5\,\upmu {\mathrm {m}}$ half a length away from the cilium. Again **a** and **b** are remarkably similar, with **c** showing at worst a 10 % error in the speed calculated by the rotlet model, and very good qualitative agreement in flow direction and magnitude

## Appendix 1: Validation of rotlet cilium models

We begin by validating the consistency of the equivalent rotlet model by considering the volume flow rate induced by a tilted rotlet located at ${\mathbf {y}}$ over a plane boundary (Blake and Chwang 1974),

15 $$\begin{aligned} u_i({\mathbf {x}}) \!=\! \frac{M_j}{8\pi \mu } \Bigg [\frac{\varepsilon _{ijk}r_k}{r^3} - \frac{\varepsilon _{ijk} R_k}{R^3} + 2h\varepsilon _{kj3} \left( \frac{\delta _{ik}}{R^3}-\frac{3R_iR_k}{R^5}\right) + 6\varepsilon _{kj3}\frac{ R_i R_k R_3}{R^5}\Bigg ], \end{aligned}$$

for ${\mathbf {r}} = {\mathbf {x}}-{\mathbf {y}}$, ${\mathbf {R}} = {\mathbf {x}}-{\mathbf {y}}^\perp$ and $R^2 = |{\mathbf {x}} - {\mathbf {y}}^\perp |^2$ with $h=y_3$ the distance between the singularity and the plane and ${\mathbf {y}}^\perp =(y_1,y_2,-y_3)$. The volume flow rate *Q* in the $\hat{{x}}_1$-direction can be found by integrating the far-field terms,

16 $$\begin{aligned} \begin{aligned} u_i^{\text{ far }}= & {} \frac{6\varepsilon _{kj3}M_jx_ix_kx_3}{|{\mathbf {x}}|^5} + \frac{6h\varepsilon _{ik3}M_3x_kx_3}{|{\mathbf {x}}|^5}, \\ Q= & {} \int \limits _{0}^{\infty } \int \limits _{-\infty }^{\infty } u_1\,{\mathrm {d}}x_2 {\mathrm {d}}x_3 = \frac{M\sin \theta }{2\pi \mu }, \end{aligned} \end{aligned}$$

from which we see only the $\hat{{x}}_2$-component arising from the tilt, $M\sin \theta$, contributes to volume flow rate in the $\hat{{x}}_1$-direction. Substituting our equivalent rotlet strength (11), we see

17 $$\begin{aligned} Q=-\frac{M\sin \theta }{2\pi \mu } = \frac{C_\perp \omega L^3\sin ^2\psi \sin \theta }{6\pi \mu }, \end{aligned}$$

which matches the formula based on the force exerted by the whirling rod (Smith et al. 2008),

18 $$\begin{aligned} Q = \frac{C_\perp \omega L^3}{6\pi \mu }\sin ^2\psi \sin \theta . \end{aligned}$$

In order to examine the accuracy of the equivalent rotlet model, we now consider a slender filament exhibiting kinematics given by Eq. (7), and examine the flow using the method of regularised stokeslets. For a cilium above a plane boundary, the velocity at a point ${\mathbf {x}}$ in the fluid is given by

19 $$\begin{aligned} {\mathbf {u}}({\mathbf {x}}) = \int _S {\mathbf {f}}({\varvec{\xi }})\cdot {\mathbf {B}}^\varepsilon ({\mathbf {x}},{\varvec{\xi }})\, {\mathrm {d}}S_{{\varvec{\xi }}}, \end{aligned}$$

where ${\varvec{\xi }}$ defines the cilium centreline and ${\mathbf {f}}({\varvec{\xi }})$ is the unknown force per unit length that the cilium exerts on the fluid. The tensor ${\mathbf {B}}^\varepsilon$ is the regularised blakelet (Ainley et al. 2008; Smith et al. 2011), which incorporates image singularities with the regularised stokeslet to enforce no slip on the plane boundary

20 $$\begin{aligned} B_{ij}^\varepsilon \left( {\mathbf {x}},{\varvec{\xi }}\right)= & {} \frac{1}{8\pi \mu }\bigg (\frac{\delta _{ij}\left( r^2 + 2\varepsilon ^2\right) + r_ir_j}{r_\varepsilon ^3} - \frac{\delta _{ij}\left( R^2 + 2\varepsilon ^2\right) + R_iR_j}{R_\varepsilon ^3} \nonumber \\&+ 2h\varDelta _{jk}\left[ \frac{\partial }{\partial R_k} \left( \frac{hR_i}{R_\varepsilon ^3} - \frac{\delta _{i3} (R^2 + 2\varepsilon ^2) + R_i R_3}{R_\varepsilon ^3} \right) - 4\pi h \delta _{ik} \phi _\varepsilon (R)\right] \nonumber \\&- \frac{6h\varepsilon ^2}{R_\varepsilon ^5}\left( \delta _{i3}R_j - \delta _{ij}R_3\right) \bigg ), \end{aligned}$$

where $\varepsilon$ is a small regularisation parameter, chosen to be the radius of the cilium. We split the cilium into 20 elements of constant force per unit length, and for each timestep collocate the known centreline velocity at the centre of each element ${\mathbf {u}}({\varvec{\xi }}_i)$. This discretisation yields the linear system

21 $$\begin{aligned} {\mathbf {u}}({\varvec{\xi }}_m) = \sum _{n=1}^N{\mathbf {f}}_n\cdot \int _{S_n} {\mathbf {B}}^\varepsilon ({\varvec{\xi }}_m,{\varvec{\xi }})\, {\mathrm {d}}S_{{\varvec{\xi }}}, \end{aligned}$$

which can be solved for the unknown force per unit length over each element. Having calculated the unknown force the cilium exerts upon the fluid at each timestep, we apply formula (19) on a grid of points in the bulk flow, time average over a single beat, and compare them to the flow generated by a plane-boundary rotlet of ‘equivalent’ strength (15).

We begin by considering a cilium of length $5\,\upmu {\mathrm {m}}$ and diameter $1/3\,\upmu {\mathrm {m}}$, beating at 30 Hz, which is typical of cilia found in KV. In the regularised blakelet model, this corresponds to a regularisation parameter of $\varepsilon = 5/30$. The semicone angle $\psi = 30^{\circ }$, and the tilt angle $\theta = 30^{\circ }$. The cilium is tilted in the *y*-direction. We calculate the strength of the equivalent rotlet using both the analytical formula (11) and the ‘volume flow rate matching’ procedure detailed in Appendix 2. We find that using a normal force coefficient of the form (Lighthill 1976)

22 $$\begin{aligned} C_\perp = \frac{8\pi \mu }{1 + 2\log (2q/a)} \end{aligned}$$

with $a = \varepsilon$ and $q = L/3$ produces a relative difference between the equivalent rotlet strength as calculated by the two methods of less than 1 % for a wide range of cilium lengths and diameters, and thus we use the analytical form in these tests.

Time-averaged flow in the plane $z = 7.5\,\upmu {\mathrm {m}}$ from the average of 30 instants over a single beat as calculated by the resolved cilium model is shown in comparison to flow generated by the equivalent rotlet in Fig. 9. Despite the fact that this plane is only half a cilium length away from the cilium tip at its zenith, the equivalent rotlet shows at worst a 10 % error in the calculated flow, and striking quantitative similarity in the flow strength and direction. Similarly, Fig. 10 shows this flow comparison in the plane $y = -2.5\,\upmu {\mathrm {m}}$, which is again half a length from the cilium, showing at worst a 10 % error in the calculated flow. Thus we conclude that even in regions relatively near to the cilium (ie greater than half a length), the equivalent rotlet provides an accurate description of the flow mechanics.

## Appendix 2: Volume flow rate-matching for more complex waveforms

Given a cilium with a more complex centreline parameterisation, for instance obtained through high-speed image microscopy, equivalent singularity cilia may also be calculated numerically by matching volume flow rates with a time-dependent slender body theory cilium model. Beats with helicity induce a net force on the fluid in the $\hat{{\mathbf {n}}}$-direction, in addition to the torque component. The volume flow rate per beat induced by a whirling cilium in the $\hat{{x}}_1$- and $\hat{{x}}_2$-directions can be matched to a plane-boundary rotlet and a blakelet (stokeslet with image singularities) using a force-per-unit length extracted from the time-dependent code,

23a $$\begin{aligned} Q_1 =&\ \left\langle \int \limits _0^L\frac{f_1(s) \xi _3(s)}{\pi \mu }\, {\mathrm {d}}s \right\rangle = \frac{M\sin {\theta }}{2\pi \mu }, \end{aligned}$$

23b $$\begin{aligned} Q_2 =&\ \left\langle \int \limits _0^L\frac{f_2(s) \xi _3(s)}{\pi \mu }\, {\mathrm {d}}s \right\rangle = \frac{hF\sin {\theta }}{\pi \mu }, \end{aligned}$$

so that

24 $$\begin{aligned} M = \frac{2}{\sin {\theta }}\left\langle {\int \limits _0^Lf_1(s) \xi _3(s)\, {\mathrm {d}}s}\right\rangle , \quad F = \frac{1}{h\sin {\theta }} \left\langle {\int \limits _0^Lf_2(s) \xi _3(s)\, {\mathrm {d}}s}\right\rangle . \end{aligned}$$

The drawback of this numerical technique is that it requires a slender body theory code, and fresh calculation of *M* and *F* for different cilium lengths and kinematics, which is not necessary with the analytical treatment above. However, an advantage is that this method accounts for non-local hydrodynamic interactions in the force calculation, and the numerical method may prove increasingly valuable as techniques for three-dimensional waveform reconstruction from image microscopy begin to deliver high-resolution kinematics (Wilson et al. 2013).
